# Supplementary material for: A Bayesian Joint Model of Multiple Nonlinear Longitudinal and Competing Risks Outcomes for Dynamic Prediction in Multiple Myeloma: Joint Estimation and Corrected Two‐Stage Approaches
Source: Stat Med. 2025 Jan 26;44(3-4):e10322. doi: 10.1002/sim.10322 (PMC11771571; doi:10.1002/sim.10322)
Supplement: Supplementary file 1 — Data S1. Supporting Information. [file SIM-44-0-s001.pdf]

SUPPLEMENTARY MATERIAL

A Bayesian joint model of multiple nonlinear longitudinal and competing risks outcomes for dynamic prediction in multiple myeloma: Joint estimation and corrected two-stage approaches

Danilo Alvares[*1], **Jessica K. Barrett**[1], **François Mercier**[2], **Spyros Roumpanis**[2], **Sean Yiu**[3], **Felipe Castro**[2], **Jochen Schulze**[2], **and Yajing Zhu**[**2]

[1]MRC Biostatistics Unit, University of Cambridge, U.K.

[2]F. Hoffmann-La Roche Ltd, Basel, Switzerland

[3]Roche Products Ltd, Welwyn Garden City, U.K.

[*]*email:* danilo.alvares@mrc-bsu.cam.ac.uk

[**]*email:* yajing.zhu@roche.com

Appendix A. Web Tables

Web Table 1: Summary (number of cases and percentage) of categorical variables by line of therapy (LoT). In terms of modelling, the first category of each variable is defined as the reference category.

**Baseline variable**	**LoT 1**	**LoT 2**	**LoT 3**	**LoT 4**
Sex				
Male	2981 (54.3%)	1536 (55.4%)	756 (54.0%)	395 (55.2%)
Female	2509 (45.7%)	1239 (44.6%)	645 (46.0%)	321 (44.8%)
Ethnicity				
Non-Hispanic white	3063 (55.8%)	1613 (58.1%)	838 (59.8%)	437 (61.0%)
Non-Hispanic black	906 (16.5%)	445 (16.0%)	217 (15.5%)	109 (15.2%)
Other	960 (17.5%)	486 (17.5%)	239 (17.1%)	114 (15.9%)
Not reported	561 (10.2%)	231 (8.3%)	107 (7.6%)	56 (7.8%)
ECOG				
0	1166 (21.2%)	597 (21.5%)	303 (21.6%)	142 (19.8%)
1	1382 (25.2%)	694 (25.0%)	358 (25.6%)	190 (26.5%)
2^+	781 (14.2%)	361 (13.0%)	163 (11.6%)	77 (10.8%)
Not reported	2161 (39.4%)	1123 (40.5%)	577 (41.2%)	307 (42.9%)
ISS				
Stage I	1144 (20.8%)	581 (20.9%)	282 (20.1%)	141 (19.7%)
Stage II	1129 (20.6%)	588 (21.2%)	299 (21.3%)	142 (19.8%)
Stage III	1141 (20.8%)	630 (22.7%)	337 (24.1%)	192 (26.8%)
Not reported	2076 (37.8%)	976 (35.2%)	483 (34.5%)	241 (33.7%)

Web Table 2: Summary of continuous variables in their original scales (Initial) and after log transformation[1], standardisation, and imputation[2] (Final). NA represents the number of missing observations before imputation.

Baseline variable	Data	Mean	SD[3]	Median	Min	Max	NA (%)
Age	Initial	67.92	10.32	69.00	24.00	84.00	–
(years)	Final	0.00	1.00	0.17	-6.18	1.36	–
Albumin	Initial	36.12	12.02	38.00	0.04	519.00	1512 (28)
(serum, g/L)	Final	0.00	0.85	0.03	-10.95	5.46	–
B2M	Initial	10.01	103.88	4.00	0.20	3800.00	3041 (55)
(serum, mg/L)	Final	0.00	0.67	0.00	-3.75	9.22	–
Creatinine	Initial	1.51	1.85	1.10	0.40	83.00	1546 (28)
(serum, mg/dL)	Final	0.00	0.85	0.00	-1.95	8.09	–
Hemoglobin	Initial	10.78	2.17	10.70	3.10	22.50	736 (13)
(g/dL)	Final	0.00	0.93	0.00	-5.87	3.67	–
LDH	Initial	230.25	195.33	179.00	50.00	3182.00	3426 (62)
(serum, U/L)	Final	0.00	0.61	0.00	-2.72	5.53	–
Lymphocyte	Initial	1.82	1.23	1.60	0.00	32.70	1614 (29)
(count, $\times 10^9$/L)	Final	0.00	0.84	0.00	-5.87	6.12	–
Neutrophil	Initial	5.93	87.97	3.50	0.00	4758.00	2252 (41)
(count, $\times 10^9$/L)	Final	0.00	0.77	0.00	-6.32	12.75	–
Platelet	Initial	227.11	93.23	217.00	0.00	921.00	1635 (30)
(count, $\times 10^9$/L)	Final	0.00	0.84	0.00	-17.22	3.35	–
IgA	Initial	7.04	14.81	0.67	0.00	136.00	2663 (49)
(serum, g/L)	Final	0.00	0.72	0.00	-1.52	2.71	–
IgG	Initial	26.39	25.36	17.09	0.40	139.01	2544 (46)
(serum, g/L)	Final	0.00	0.73	0.00	-3.01	1.94	–
IgM	Initial	0.51	3.11	0.24	0.00	97.43	3105 (57)
(serum, g/L)	Final	0.00	0.66	0.00	-1.95	8.49	–

[1] $\log(x + 0.1)$ to avoid numerical problems when $x = 0$.
[2] Mean-value imputation strategy for missing values (Donders et al., 2006), i.e., we replace them with zeros (after standardisation, the mean of each variable is zero).
[3] After standardisation, the standard deviation is equal to 1, but the imputation concentrates more values at zero and consequently reduces such standard deviation.

References

Donders, A. R. T., van der Heijden, G. J. M. G., Stijnen, T., and Moons, K. G. M. (2006). Review: A gentle introduction to imputation of missing values. *Journal of Clinical Epidemiology* **59,** 1087–1091.

Kerioui, M., Bertrand, J., Bruno, R., Mercier, F., Guedj, J., and Desmée, S. (2022). Modelling the association between biomarkers and clinical outcome: An introduction to nonlinear joint models. *British Journal of Clinical Pharmacology* **88,** 1452–1463.

Web Table 3: Posterior summary (mean and 95% credible interval) of the bi-exponential submodel parameters for each biomarker by line of therapy (LoT) using the joint estimation (JE) approach. Statistically significant variables are shown in bold, except for variance parameters.

**Interpretation**	**Parameter**	**LoT $l = 1$**	**LoT $l = 2$**	**LoT $l = 3$**	**LoT $l = 4$**
M-spike ($k = 1$)					
Baseline	$\exp\{\theta_{1kl}\}$	**17.086** (16.405, 17.797)	**7.765** (7.347, 8.232)	**7.382** (6.780, 8.057)	**7.950** (7.030, 8.971)
Growth	$\exp\{\theta_{2kl}\}$	**0.246** (0.234, 0.259)	**0.293** (0.257, 0.332)	**0.377** (0.311, 0.450)	**0.235** (0.158, 0.331)
Decay	$\exp\{\theta_{3kl}\}$	**4.056** (3.815, 4.300)	1.092 (0.918, 1.295)	1.200 (0.921, 1.505)	0.924 (0.621, 1.327)
Residual error variance	σ^2_{kl}	0.053 (0.051, 0.054)	0.050 (0.048, 0.052)	0.061 (0.057, 0.064)	0.053 (0.049, 0.057)
Covariance matrix for random effects	ω_{11kl}	0.865 (0.806, 0.928)	0.922 (0.841, 1.011)	1.025 (0.903, 1.160)	1.033 (0.866, 1.231)
	ω_{12kl}	**-0.146** (-0.197, -0.094)	0.022 (-0.083, 0.128)	-0.160 (-0.325, 0.001)	-0.160 (-0.476, 0.150)
	ω_{13kl}	**0.595** (0.521, 0.672)	**0.776** (0.621, 0.946)	**0.701** (0.489, 0.933)	**0.655** (0.314, 1.052)
	ω_{22kl}	0.709 (0.641, 0.781)	1.134 (0.966, 1.328)	1.182 (0.961, 1.455)	1.994 (1.401, 2.842)
	ω_{23kl}	0.044 (-0.017, 0.106)	**0.755** (0.549, 0.981)	**0.385** (0.100, 0.708)	**1.424** (0.770, 2.285)
	ω_{33kl}	1.229 (1.114, 1.350)	2.621 (2.184, 3.116)	1.977 (1.509, 2.567)	2.986 (2.048, 4.321)
Free light chains ($k = 2$)					
Baseline	$\exp\{\theta_{1kl}\}$	**20.748** (19.340, 22.189)	**9.381** (8.662, 10.178)	**10.246** (9.075, 11.575)	**14.002** (11.646, 17.098)
Growth	$\exp\{\theta_{2kl}\}$	**0.162** (0.149, 0.177)	**0.342** (0.300, 0.388)	**0.426** (0.350, 0.518)	**0.392** (0.284, 0.517)
Decay	$\exp\{\theta_{3kl}\}$	**2.903** (2.651, 3.164)	0.916 (0.736, 1.121)	1.109 (0.797, 1.499)	0.997 (0.590, 1.589)
Residual error variance	σ^2_{kl}	0.100 (0.097, 0.102)	0.075 (0.072, 0.077)	0.080 (0.076, 0.083)	0.102 (0.095, 0.109)
Covariance matrix for random effects	ω_{11kl}	3.017 (2.841, 3.198)	2.330 (2.150, 2.524)	2.525 (2.248, 2.827)	2.792 (2.375, 3.275)
	ω_{12kl}	**-1.199** (-1.332, -1.072)	0.076 (-0.083, 0.234)	0.247 (-0.023, 0.518)	-0.052 (-0.464, 0.358)
	ω_{13kl}	**1.914** (1.749, 2.095)	**1.874** (1.616, 2.148)	**1.851** (1.463, 2.289)	**2.099** (1.453, 2.872)
	ω_{22kl}	1.477 (1.340, 1.623)	1.928 (1.701, 2.185)	2.335 (1.945, 2.791)	2.263 (1.725, 2.941)
	ω_{23kl}	0.038 (-0.105, 0.183)	**1.354** (1.055, 1.683)	**1.582** (1.102, 2.144)	**1.025** (0.293, 1.875)
	ω_{33kl}	2.980 (2.726, 3.268)	4.473 (3.821, 5.210)	4.364 (3.415, 5.525)	5.008 (3.518, 7.075)

Web Table 4: Posterior summary (mean and 95% credible interval) of the bi-exponential submodel parameters for each biomarker by line of therapy (LoT) using the corrected two-stage (TS) approach. Statistically significant variables are shown in bold, except for variance parameters.

**Interpretation**	**Parameter**	**LoT $l = 1$**	**LoT $l = 2$**	**LoT $l = 3$**	**LoT $l = 4$**
		M-spike ($k = 1$)			
Baseline	$\exp\{\theta_{1kl}\}$	**16.841** (16.197, 17.546)	**7.675** (7.250, 8.160)	**7.388** (6.792, 8.047)	**7.786** (6.920, 8.759)
Growth	$\exp\{\theta_{2kl}\}$	**0.196** (0.185, 0.207)	**0.220** (0.191, 0.249)	**0.283** (0.231, 0.344)	**0.215** (0.139, 0.306)
Decay	$\exp\{\theta_{3kl}\}$	**3.624** (3.401, 3.858)	0.910 (0.763, 1.080)	1.049 (0.827, 1.310)	0.874 (0.562, 1.283)
Residual error variance	σ^2_{kl}	0.053 (0.051, 0.054)	0.051 (0.049, 0.053)	0.061 (0.058, 0.064)	0.053 (0.049, 0.057)
Covariance matrix for random effects	ω_{11kl}	0.865 (0.808, 0.927)	0.917 (0.838, 1.004)	1.013 (0.894, 1.146)	1.030 (0.860, 1.232)
	ω_{12kl}	**-0.135** (-0.193, -0.079)	-0.035 (-0.162, 0.087)	**-0.206** (-0.389, -0.024)	-0.165 (-0.485, 0.147)
	ω_{13kl}	**0.631** (0.554, 0.711)	**0.766** (0.604, 0.941)	**0.672** (0.452, 0.909)	**0.663** (0.316, 1.063)
	ω_{22kl}	0.661 (0.592, 0.737)	1.292 (1.079, 1.537)	1.358 (1.066, 1.705)	2.031 (1.407, 2.931)
	ω_{23kl}	**0.196** (0.124, 0.272)	**0.981** (0.735, 1.258)	**0.595** (0.265, 0.977)	**1.456** (0.763, 2.399)
	ω_{33kl}	1.422 (1.294, 1.562)	2.911 (2.449, 3.449)	2.173 (1.657, 2.793)	3.028 (2.018, 4.394)
		Free light chains ($k = 2$)			
Baseline	$\exp\{\theta_{1kl}\}$	**19.802** (18.528, 21.254)	**8.915** (8.184, 9.663)	**9.976** (8.779, 11.311)	**13.173** (10.983, 15.915)
Growth	$\exp\{\theta_{2kl}\}$	**0.143** (0.132, 0.155)	**0.178** (0.148, 0.210)	**0.196** (0.142, 0.260)	**0.283** (0.198, 0.381)
Decay	$\exp\{\theta_{3kl}\}$	**2.605** (2.374, 2.850)	**0.506** (0.400, 0.631)	**0.642** (0.445, 0.885)	0.767 (0.431, 1.222)
Residual error variance	σ^2_{kl}	0.100 (0.098, 0.102)	0.075 (0.073, 0.078)	0.080 (0.077, 0.084)	0.102 (0.095, 0.109)
Covariance matrix for random effects	ω_{11kl}	2.977 (2.810, 3.154)	2.318 (2.139, 2.511)	2.546 (2.277, 2.842)	2.782 (2.365, 3.259)
	ω_{12kl}	**-1.234** (-1.365, -1.105)	-0.106 (-0.333, 0.113)	-0.028 (-0.461, 0.378)	-0.230 (-0.681, 0.211)
	ω_{13kl}	**1.897** (1.729, 2.076)	**1.905** (1.610, 2.223)	**1.906** (1.446, 2.408)	**2.053** (1.393, 2.826)
	ω_{22kl}	1.586 (1.438, 1.741)	2.464 (2.099, 2.905)	3.177 (2.494, 4.031)	2.432 (1.809, 3.270)
	ω_{23kl}	**0.144** (0.003, 0.290)	**2.031** (1.571, 2.575)	**2.547** (1.765, 3.531)	**1.189** (0.395, 2.155)
	ω_{33kl}	3.053 (2.787, 3.334)	5.569 (4.730, 6.598)	5.713 (4.383, 7.398)	5.319 (3.698, 7.546)

Web Table 5: Posterior summary (mean and 95% credible interval) for time-to-death ($v = 1$) by line of therapy (LoT) using the joint estimation (JE) approach. Statistically significant variables are shown in bold.

**Variable**	**Category**	**LoT $l = 1$**	**LoT $l = 2$**	**LoT $l = 3$**	**LoT $l = 4$**
Sex	Female	-0.159 (-0.323, 0.006)	**-0.339** (-0.579, -0.096)	-0.165 (-0.465, 0.134)	-0.307 (-0.627, 0.018)
Ethnicity	Non-Hisp. Black	-0.082 (-0.315, 0.143)	0.004 (-0.326, 0.326)	0.017 (-0.406, 0.426)	0.208 (-0.208, 0.602)
	Other	-0.012 (-0.237, 0.200)	-0.233 (-0.553, 0.071)	-0.355 (-0.779, 0.050)	**-0.653** (-1.148, -0.177)
ECOG	1	0.174 (-0.093, 0.446)	0.051 (-0.320, 0.423)	-0.069 (-0.517, 0.379)	**0.783** (0.321, 1.271)
	2^+	**0.806** (0.539, 1.086)	**0.665** (0.285, 1.050)	**0.610** (0.120, 1.107)	**0.963** (0.358, 1.566)
ISS	Stage II	**0.543** (0.207, 0.877)	0.197 (-0.227, 0.619)	-0.059 (-0.570, 0.471)	**0.617** (0.090, 1.162)
	Stage III	**0.464** (0.072, 0.858)	0.316 (-0.162, 0.797)	0.224 (-0.385, 0.849)	0.584 (-0.022, 1.214)
Age	–	**0.557** (0.448, 0.671)	**0.472** (0.322, 0.624)	**0.560** (0.373, 0.755)	**0.171** (0.019, 0.327)
Albumin	–	-0.073 (-0.163, 0.023)	0.056 (-0.125, 0.265)	-0.167 (-0.347, 0.030)	-0.078 (-0.320, 0.205)
B2M	–	**0.244** (0.082, 0.397)	-0.005 (-0.227, 0.200)	-0.268 (-0.588, 0.029)	0.292 (-0.043, 0.623)
Creatine	–	0.036 (-0.064, 0.134)	0.053 (-0.099, 0.200)	0.055 (-0.152, 0.257)	-0.081 (-0.314, 0.148)
Hemoglobin	–	**-0.125** (-0.220, -0.029)	-0.074 (-0.205, 0.059)	-0.131 (-0.297, 0.034)	0.091 (-0.089, 0.279)
LDH	–	**0.153** (0.041, 0.263)	0.140 (-0.025, 0.296)	0.212 (-0.013, 0.420)	-0.038 (-0.262, 0.173)
Lymphocyte	–	-0.045 (-0.134, 0.045)	-0.030 (-0.166, 0.104)	-0.017 (-0.170, 0.138)	-0.056 (-0.230, 0.121)
Neutrophil	–	**0.169** (0.061, 0.278)	**0.178** (0.018, 0.332)	0.019 (-0.166, 0.211)	0.165 (-0.049, 0.380)
Platelet	–	**-0.091** (-0.162, -0.011)	-0.080 (-0.232, 0.072)	-0.178 (-0.357, 0.008)	**-0.318** (-0.513, -0.125)
IgA	–	0.015 (-0.125, 0.157)	**0.279** (0.089, 0.469)	0.158 (-0.076, 0.389)	-0.135 (-0.361, 0.093)
IgG	–	0.129 (-0.010, 0.269)	0.172 (-0.033, 0.376)	-0.024 (-0.267, 0.221)	**-0.273** (-0.492, -0.050)
IgM	–	**0.163** (0.043, 0.279)	0.078 (-0.091, 0.227)	-0.076 (-0.324, 0.147)	-0.116 (-0.310, 0.082)
Time prev. LoT	–	–	-0.043 (-0.169, 0.078)	**-0.188** (-0.388, -0.004)	**-0.298** (-0.578, -0.039)
Baseline M-spike (α_{1lk1})	–	**0.206** (0.055, 0.359)	**0.297** (0.105, 0.490)	0.134 (-0.127, 0.397)	**0.450** (0.146, 0.778)
Growth M-spike (α_{1lk2})	–	**0.382** (0.197, 0.566)	-0.049 (-0.293, 0.201)	0.198 (-0.142, 0.530)	0.240 (-0.181, 0.680)
Decay M-spike (α_{1lk3})	–	-0.014 (-0.145, 0.116)	-0.036 (-0.191, 0.117)	0.053 (-0.201, 0.304)	-0.111 (-0.445, 0.205)
Baseline FLC (α_{2lk1})	–	0.103 (-0.035, 0.236)	**0.290** (0.162, 0.413)	**0.253** (0.077, 0.428)	**0.221** (0.055, 0.390)
Growth FLC (α_{2lk2})	–	0.107 (-0.069, 0.273)	**0.476** (0.307, 0.641)	0.164 (-0.054, 0.383)	**0.413** (0.212, 0.609)
Decay FLC (α_{2lk3})	–	0.099 (-0.014, 0.215)	-0.001 (-0.131, 0.135)	-0.070 (-0.263, 0.117)	0.017 (-0.162, 0.195)

Web Table 6: Posterior summary (mean and 95% credible interval) for time-to-death ($v = 1$) by line of therapy (LoT) using the corrected two-stage (TS) approach. Statistically significant variables are shown in bold.

Variable	Category	LoT $l = 1$	LoT $l = 2$	LoT $l = 3$	LoT $l = 4$
Sex	Female	-0.155 (-0.316, 0.004)	**-0.315** (-0.547, -0.078)	-0.152 (-0.438, 0.143)	-0.290 (-0.598, 0.017)
Ethnicity	Non-Hisp. Black	-0.087 (-0.315, 0.152)	-0.009 (-0.319, 0.314)	0.041 (-0.382, 0.451)	0.197 (-0.195, 0.588)
	Other	0.002 (-0.210, 0.214)	-0.235 (-0.548, 0.082)	-0.356 (-0.753, 0.039)	**-0.619** (-1.129, -0.144)
ECOG	1	0.158 (-0.105, 0.452)	0.052 (-0.311, 0.438)	-0.039 (-0.519, 0.413)	**0.768** (0.344, 1.253)
	2^+	**0.789** (0.523, 1.069)	**0.658** (0.290, 1.064)	**0.647** (0.134, 1.158)	**0.933** (0.359, 1.519)
ISS	Stage II	**0.545** (0.216, 0.888)	0.209 (-0.204, 0.625)	-0.034 (-0.532, 0.490)	**0.588** (0.050, 1.139)
	Stage III	**0.472** (0.075, 0.874)	0.368 (-0.101, 0.847)	0.238 (-0.381, 0.870)	0.598 (-0.022, 1.206)
Age	–	**0.555** (0.443, 0.665)	**0.457** (0.307, 0.605)	**0.572** (0.385, 0.771)	**0.163** (0.005, 0.327)
Albumin	–	-0.067 (-0.152, 0.026)	0.046 (-0.133, 0.245)	-0.165 (-0.347, 0.051)	-0.079 (-0.320, 0.209)
B2M	–	**0.239** (0.075, 0.391)	-0.002 (-0.211, 0.198)	-0.292 (-0.597, 0.003)	0.284 (-0.036, 0.610)
Creatine	–	0.041 (-0.058, 0.136)	0.050 (-0.088, 0.190)	0.054 (-0.151, 0.252)	-0.100 (-0.323, 0.110)
Hemoglobin	–	**-0.123** (-0.211, -0.027)	-0.075 (-0.218, 0.059)	-0.133 (-0.305, 0.038)	0.071 (-0.105, 0.254)
LDH	–	**0.161** (0.046, 0.274)	0.145 (-0.016, 0.292)	0.217 (-0.007, 0.420)	-0.037 (-0.262, 0.170)
Lymphocyte	–	-0.040 (-0.125, 0.044)	-0.001 (-0.123, 0.131)	-0.019 (-0.159, 0.131)	-0.066 (-0.228, 0.110)
Neutrophil	–	**0.168** (0.067, 0.272)	**0.178** (0.022, 0.333)	0.027 (-0.155, 0.206)	0.169 (-0.040, 0.367)
Platelet	–	**-0.093** (-0.161, -0.013)	-0.105 (-0.250, 0.050)	**-0.206** (-0.401, -0.007)	**-0.320** (-0.509, -0.133)
IgA	–	0.020 (-0.121, 0.165)	**0.283** (0.098, 0.468)	0.171 (-0.063, 0.405)	-0.126 (-0.344, 0.104)
IgG	–	0.120 (-0.016, 0.256)	0.179 (-0.018, 0.374)	-0.011 (-0.261, 0.237)	**-0.267** (-0.486, -0.043)
IgM	–	**0.162** (0.042, 0.276)	0.088 (-0.070, 0.242)	-0.081 (-0.332, 0.150)	-0.117 (-0.294, 0.066)
Time prev. LoT	–	–	-0.027 (-0.143, 0.084)	-0.182 (-0.384, 0.003)	**-0.300** (-0.580, -0.064)
Baseline M-spike (α_{1lk1})	–	**0.197** (0.030, 0.350)	**0.308** (0.100, 0.510)	0.140 (-0.116, 0.403)	**0.466** (0.172, 0.758)
Growth M-spike (α_{1lk2})	–	**0.245** (0.041, 0.438)	-0.065 (-0.298, 0.198)	0.123 (-0.180, 0.425)	0.266 (-0.093, 0.658)
Decay M-spike (α_{1lk3})	–	-0.046 (-0.177, 0.087)	-0.031 (-0.181, 0.125)	0.013 (-0.216, 0.251)	-0.127 (-0.440, 0.161)
Baseline FLC (α_{2lk1})	–	0.080 (-0.057, 0.218)	**0.309** (0.176, 0.443)	**0.236** (0.058, 0.419)	**0.240** (0.085, 0.400)
Growth FLC (α_{2lk2})	–	0.061 (-0.111, 0.231)	**0.282** (0.111, 0.440)	0.004 (-0.207, 0.225)	**0.330** (0.152, 0.505)
Decay FLC (α_{2lk3})	–	0.098 (-0.021, 0.216)	-0.045 (-0.168, 0.081)	-0.055 (-0.241, 0.123)	-0.028 (-0.185, 0.136)

Web Table 7: Posterior summary (mean and 95% credible interval) for time-to-next-LoT ($v = 2$) by line of therapy (LoT) using the joint estimation (JE) approach. Statistically significant variables are shown in bold.

**Variable**	**Category**	**LoT $l = 1$**	**LoT $l = 2$**	**LoT $l = 3$**
Sex	Female	-0.027 (-0.122, 0.069)	0.005 (-0.130, 0.140)	-0.185 (-0.379, 0.007)
Ethnicity	Non-Hisp. Black	**-0.220** (-0.358, -0.086)	-0.109 (-0.297, 0.076)	-0.151 (-0.422, 0.110)
	Other	-0.050 (-0.180, 0.076)	**-0.195** (-0.374, -0.014)	**-0.454** (-0.727, -0.183)
ECOG	1	0.125 (-0.010, 0.263)	0.094 (-0.096, 0.286)	0.058 (-0.219, 0.341)
	2^+	0.161 (-0.008, 0.327)	-0.094 (-0.336, 0.145)	0.125 (-0.236, 0.487)
ISS	Stage II	-0.027 (-0.179, 0.126)	0.048 (-0.163, 0.260)	-0.125 (-0.437, 0.189)
	Stage III	0.090 (-0.102, 0.285)	0.048 (-0.203, 0.302)	0.175 (-0.190, 0.534)
Age	–	**-0.210** (-0.256, -0.164)	**-0.124** (-0.187, -0.063)	**-0.168** (-0.259, -0.076)
Albumin	–	0.005 (-0.054, 0.067)	-0.050 (-0.146, 0.053)	-0.034 (-0.188, 0.132)
B2M	–	0.068 (-0.035, 0.168)	-0.040 (-0.172, 0.089)	-0.098 (-0.278, 0.078)
Creatine	–	-0.028 (-0.096, 0.037)	-0.048 (-0.144, 0.045)	-0.011 (-0.148, 0.122)
Hemoglobin	–	**-0.067** (-0.125, -0.010)	-0.002 (-0.080, 0.074)	0.020 (-0.095, 0.134)
LDH	–	-0.065 (-0.140, 0.009)	-0.047 (-0.152, 0.058)	0.019 (-0.137, 0.173)
Lymphocyte	–	0.035 (-0.021, 0.091)	-0.043 (-0.125, 0.039)	-0.009 (-0.114, 0.096)
Neutrophil	–	-0.054 (-0.118, 0.011)	-0.015 (-0.106, 0.075)	-0.075 (-0.198, 0.051)
Platelet	–	-0.008 (-0.061, 0.047)	-0.047 (-0.138, 0.044)	-0.075 (-0.196, 0.046)
IgA	–	**-0.101** (-0.178, -0.025)	0.009 (-0.104, 0.116)	-0.043 (-0.185, 0.099)
IgG	–	**-0.107** (-0.184, -0.030)	-0.045 (-0.155, 0.065)	**-0.174** (-0.319, -0.030)
IgM	–	-0.064 (-0.139, 0.010)	-0.039 (-0.141, 0.056)	-0.099 (-0.244, 0.038)
Time prev. LoT	–	–	**-0.113** (-0.190, -0.038)	**-0.166** (-0.286, -0.049)
Baseline M-spike (α_{1lk1})	–	**0.332** (0.233, 0.430)	**0.311** (0.186, 0.437)	**0.480** (0.299, 0.663)
Growth M-spike (α_{1lk2})	–	**0.971** (0.855, 1.086)	**0.631** (0.444, 0.814)	**0.569** (0.375, 0.769)
Decay M-spike (α_{1lk3})	–	0.036 (-0.049, 0.122)	-0.070 (-0.180, 0.041)	**-0.205** (-0.379, -0.035)
Baseline FLC (α_{2lk1})	–	**0.277** (0.199, 0.354)	**0.206** (0.122, 0.290)	**0.256** (0.139, 0.376)
Growth FLC (α_{2lk2})	–	**0.295** (0.183, 0.405)	**0.483** (0.369, 0.599)	**0.611** (0.468, 0.759)
Decay FLC (α_{2lk3})	–	-0.028 (-0.096, 0.040)	0.080 (-0.012, 0.173)	0.041 (-0.095, 0.179)

Web Table 8: Posterior summary (mean and 95% credible interval) for time-to-next-LoT ($v = 2$) by line of therapy (LoT) using the corrected two-stage (TS) approach. Statistically significant variables are shown in bold.

**Variable**	**Category**	**LoT $l = 1$**	**LoT $l = 2$**	**LoT $l = 3$**
Sex	Female	-0.027 (-0.119, 0.063)	0.015 (-0.113, 0.140)	**-0.195** (-0.381, -0.004)
Ethnicity	Non-Hisp. Black	**-0.220** (-0.361, -0.092)	-0.127 (-0.327, 0.054)	-0.119 (-0.386, 0.139)
	Other	-0.021 (-0.152, 0.094)	**-0.211** (-0.386, -0.042)	**-0.494** (-0.767, -0.222)
ECOG	1	0.117 (-0.026, 0.256)	0.107 (-0.073, 0.284)	0.076 (-0.203, 0.351)
	2^+	0.152 (-0.022, 0.321)	-0.079 (-0.313, 0.144)	0.119 (-0.240, 0.469)
ISS	Stage II	-0.002 (-0.152, 0.153)	0.051 (-0.159, 0.245)	-0.056 (-0.333, 0.244)
	Stage III	0.101 (-0.079, 0.288)	0.094 (-0.148, 0.335)	0.210 (-0.149, 0.568)
Age	–	**-0.220** (-0.262, -0.178)	**-0.141** (-0.200, -0.080)	**-0.171** (-0.254, -0.087)
Albumin	–	0.009 (-0.047, 0.067)	-0.057 (-0.147, 0.038)	-0.055 (-0.206, 0.101)
B2M	–	0.062 (-0.033, 0.154)	-0.035 (-0.165, 0.082)	-0.123 (-0.305, 0.043)
Creatine	–	-0.019 (-0.083, 0.045)	-0.051 (-0.145, 0.041)	-0.003 (-0.140, 0.122)
Hemoglobin	–	-0.056 (-0.111, 0.003)	-0.001 (-0.073, 0.070)	0.019 (-0.091, 0.125)
LDH	–	-0.044 (-0.113, 0.026)	-0.053 (-0.157, 0.042)	0.023 (-0.118, 0.164)
Lymphocyte	–	0.035 (-0.021, 0.086)	-0.025 (-0.104, 0.052)	-0.013 (-0.120, 0.091)
Neutrophil	–	-0.050 (-0.111, 0.011)	-0.010 (-0.100, 0.080)	-0.061 (-0.183, 0.066)
Platelet	–	-0.012 (-0.061, 0.038)	-0.067 (-0.157, 0.021)	-0.099 (-0.220, 0.021)
IgA	–	**-0.098** (-0.178, -0.018)	0.009 (-0.098, 0.115)	-0.027 (-0.170, 0.117)
IgG	–	**-0.122** (-0.202, -0.045)	-0.044 (-0.149, 0.058)	**-0.168** (-0.321, -0.013)
IgM	–	-0.063 (-0.138, 0.013)	-0.031 (-0.132, 0.067)	-0.099 (-0.237, 0.033)
Time prev. LoT	–	–	**-0.107** (-0.185, -0.029)	**-0.164** (-0.295, -0.041)
Baseline M-spike (α_{1lk1})	–	**0.376** (0.288, 0.473)	**0.367** (0.252, 0.488)	**0.507** (0.340, 0.681)
Growth M-spike (α_{1lk2})	–	**0.803** (0.692, 0.918)	**0.561** (0.404, 0.724)	**0.520** (0.338, 0.721)
Decay M-spike (α_{1lk3})	–	-0.063 (-0.137, 0.009)	-0.084 (-0.182, 0.016)	**-0.229** (-0.386, -0.084)
Baseline FLC (α_{2lk1})	–	**0.269** (0.192, 0.347)	**0.253** (0.173, 0.337)	**0.357** (0.243, 0.475)
Growth FLC (α_{2lk2})	–	**0.243** (0.148, 0.337)	**0.320** (0.220, 0.417)	**0.452** (0.313, 0.594)
Decay FLC (α_{2lk3})	–	-0.037 (-0.105, 0.029)	0.005 (-0.077, 0.084)	-0.069 (-0.185, 0.047)

Web Table 9: Baseline variables of patients used to illustrate individual dynamic predictions.

**Baseline variable**	**Patient A**	**Patient B**
Sex	Male	Female
Ethnicity	Non-Hispanic white	Non-Hispanic white
ECOG	2^+	0
ISS	Not reported	Stage III
Age (years)	72	73
Albumin (serum, g/L)	34	19
B2B (serum, mg/L)	NA	13.8
Creatinine (serum, mg/dL)	0.93	0.9
Hemoglobin (g/dL)	13.8	9.9
LDH (serum, U/L)	Not reported	308
Lymphocyte (count, $\times 10^9$/L)	0.847	1.5
Neutrophil (count, $\times 10^9$/L)	4.7	5.4
Platelet (count, $\times 10^9$/L)	233	215
IgA (serum, g/L)	NA	0.18
IgG (serum, g/L)	83	93.68
IgM (serum, g/L)	NA	0.15

Appendix B. Web Figures

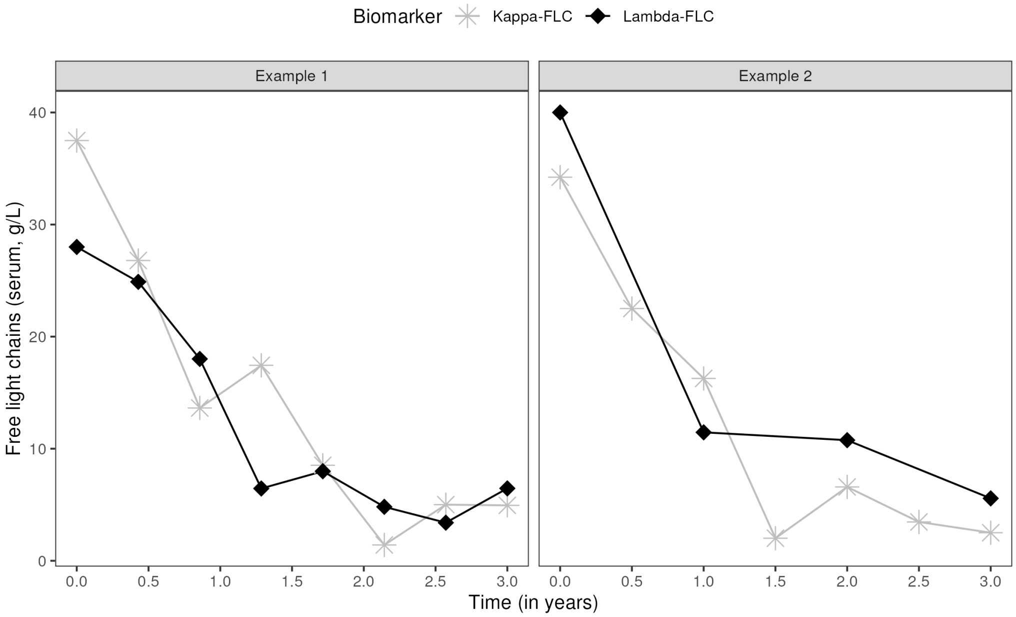

Web Figure 1: Illustrations of kappa and lambda free light chain (FLC) trajectories. In Example 1 (left), as the first kappa-FLC value is higher than the first lambda-FLC value, only kappa-FLC values are followed through. In Example 2 (right) the opposite occurs, as the first lambda-FLC value is higher than the first kappa-FLC value, only lambda-FLC values are followed through.

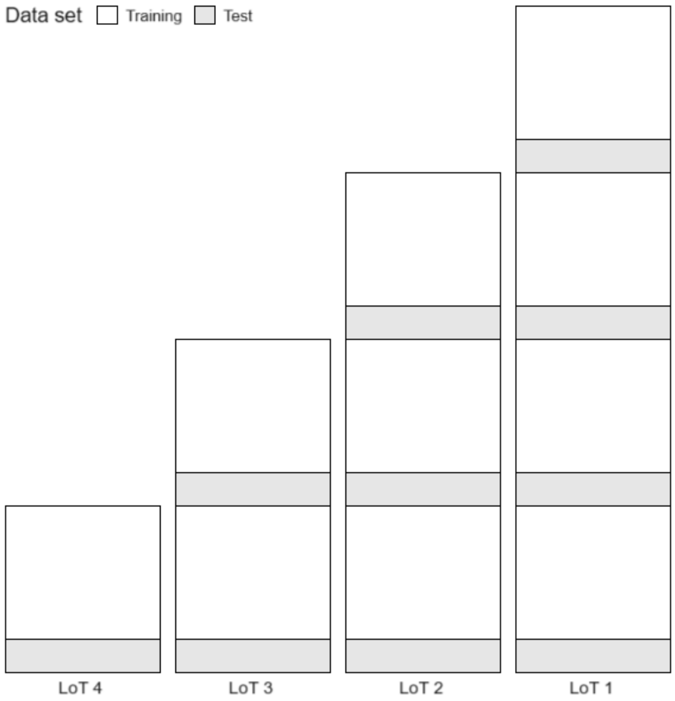

Web Figure 2: Data splitting scheme into training and test sets by line of therapy (LoT). From bottom to top, first row represents patients in LoTs 1-4, and last one represents patients in LoT 1 but not in LoTs 2-4.

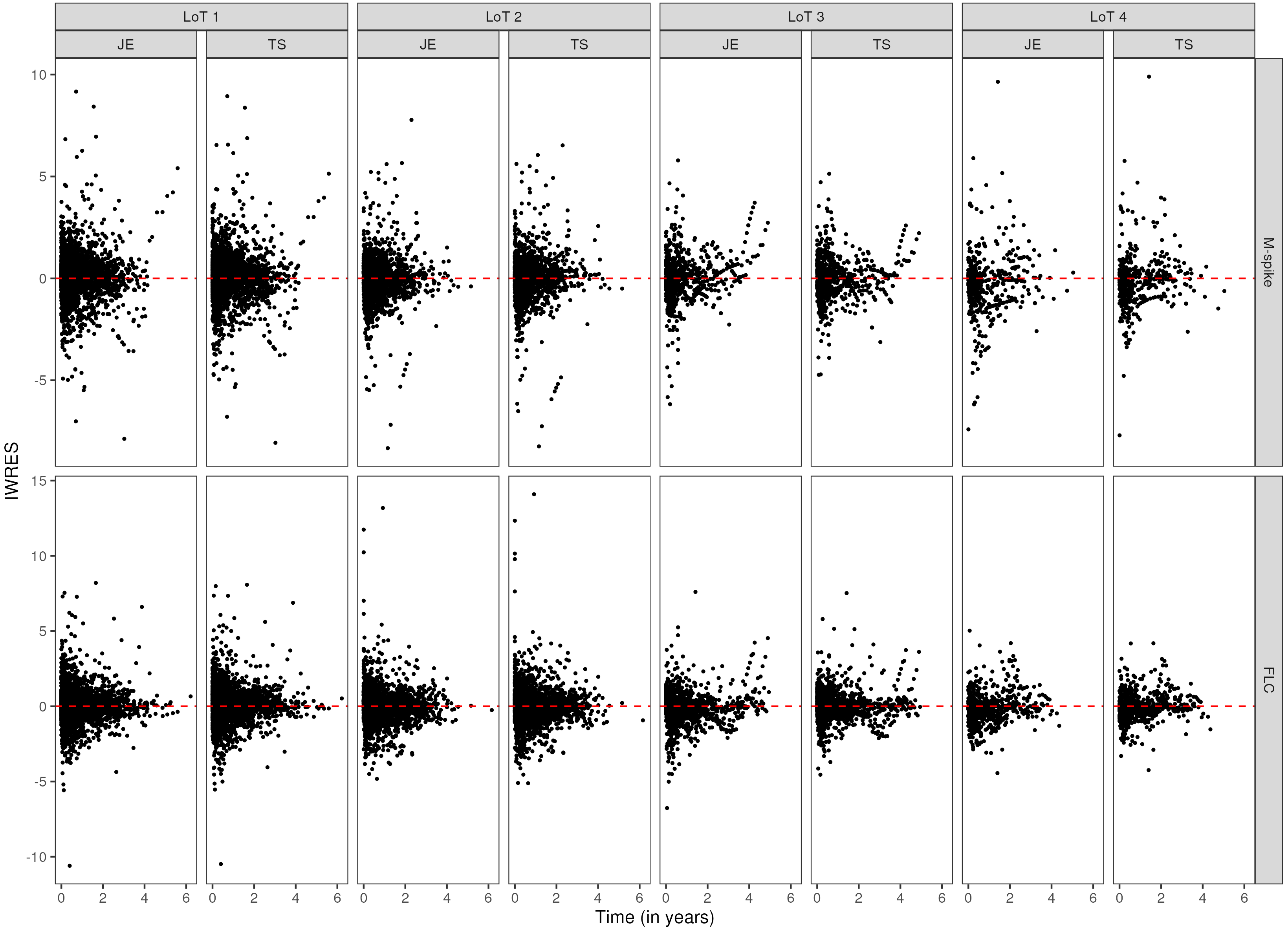

Web Figure 3: Individual weighted residuals (IWRES) from the bi-exponential submodel for each biomarker considering the test data set by line of therapy (LoT) using joint estimation (JE) and corrected two-stage (TS) approaches. For suitable fit, IWRES should approximately follow a Normal distribution centred at zero (Kerioui et al., 2022).

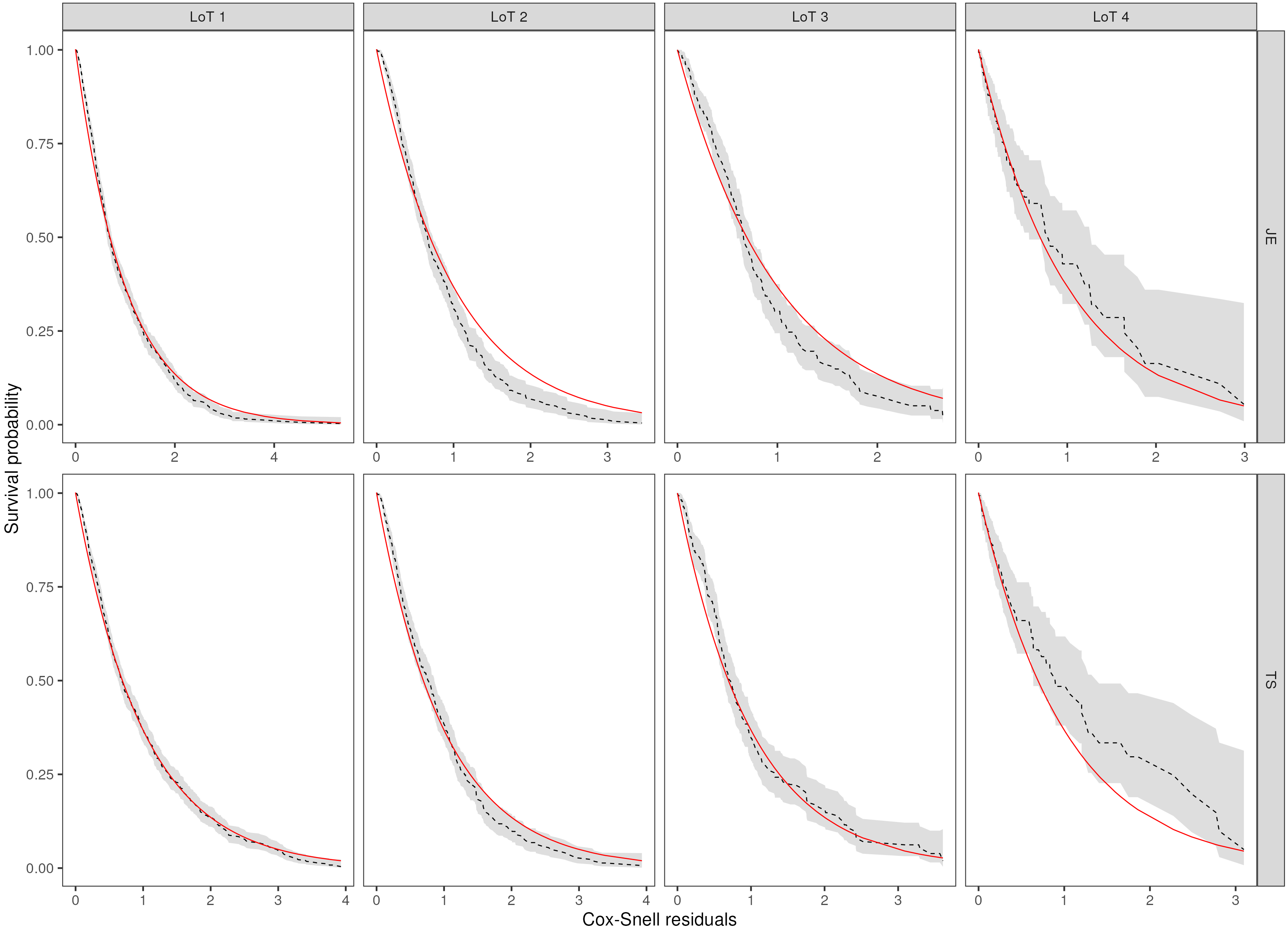

Web Figure 4: Kaplan–Meier estimates of the Cox–Snell residuals (dashed black line) and its 95% confidence interval (gray shadow) from the survival submodel considering the test data set by line of therapy (LoT) using joint estimation (JE) and corrected two-stage (TS) approaches. Solid red line: the survival function of the unit exponential distribution.

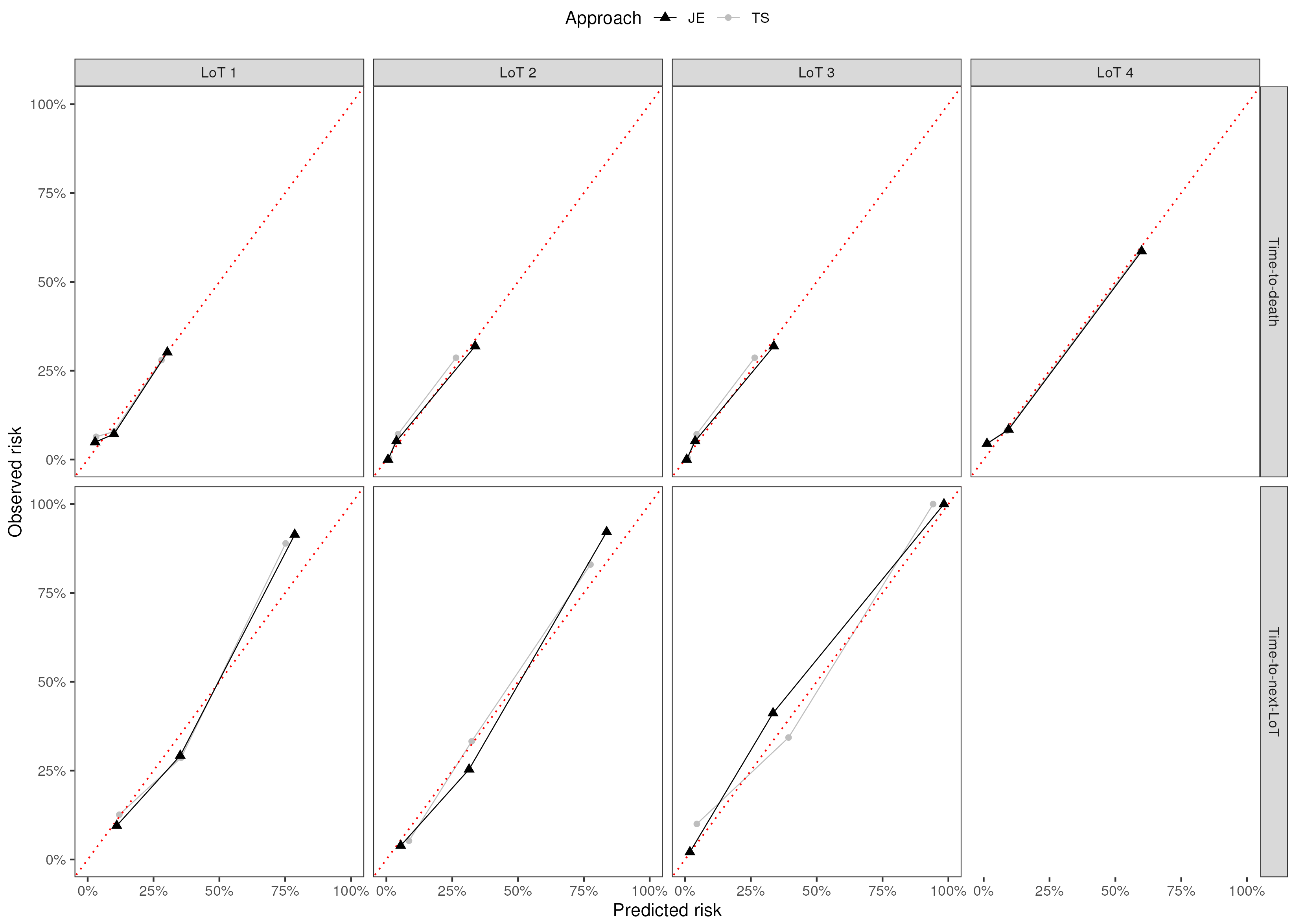

Web Figure 5: Calibration plots by tercile of predicted 1-year risk from the survival submodel with 6 months of landmark time for each time-to-event considering the test data set by line of therapy (LoT) using joint estimation (JE, black triangle) and corrected two-stage (TS, gray circle) approaches.

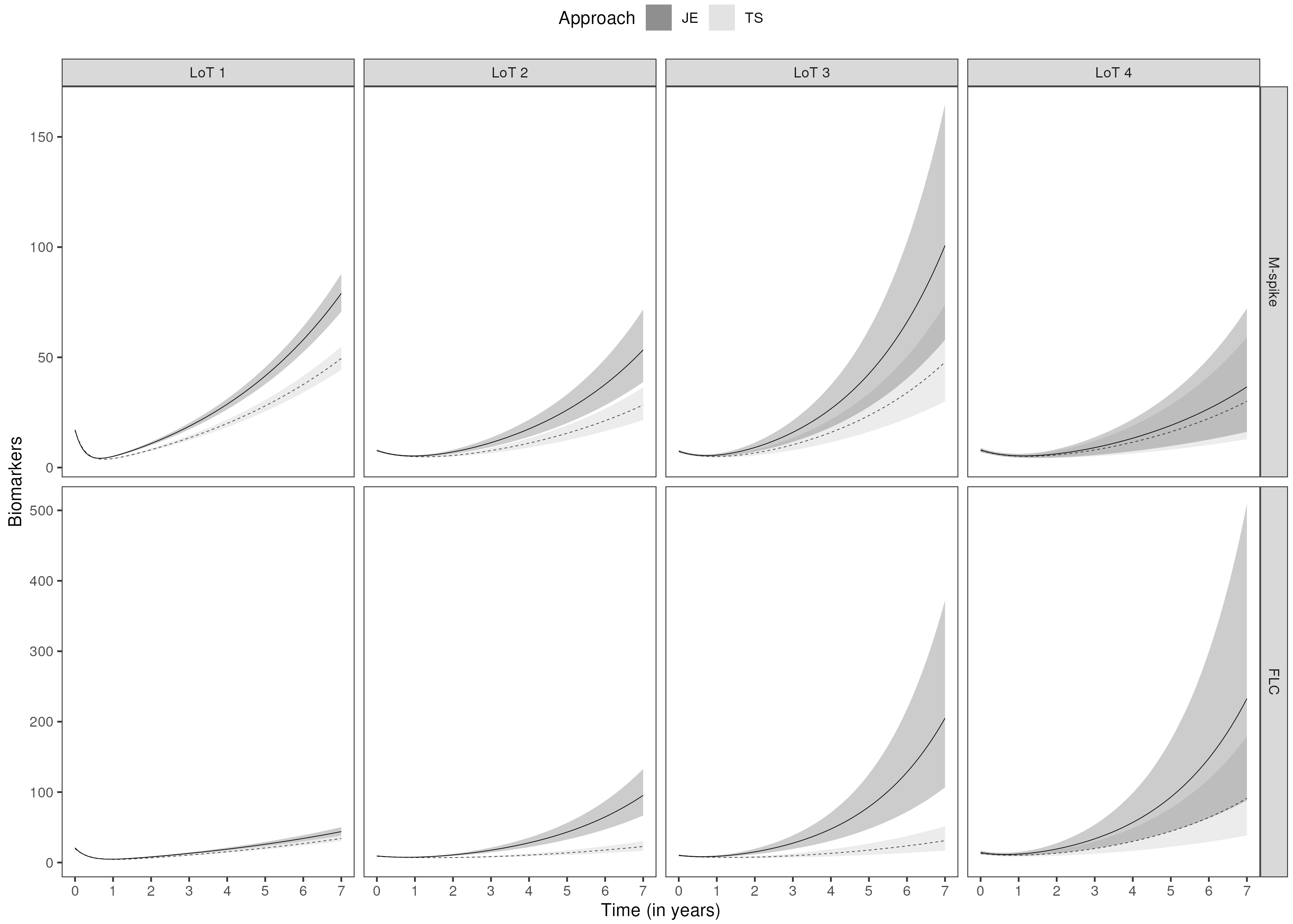

Web Figure 6: Posterior mean trajectory and its 95% credible interval from the bi-exponential submodel for each biomarker by line of therapy (LoT) using joint estimation (JE, solid line) and corrected two-stage (TS, dashed line) approaches.

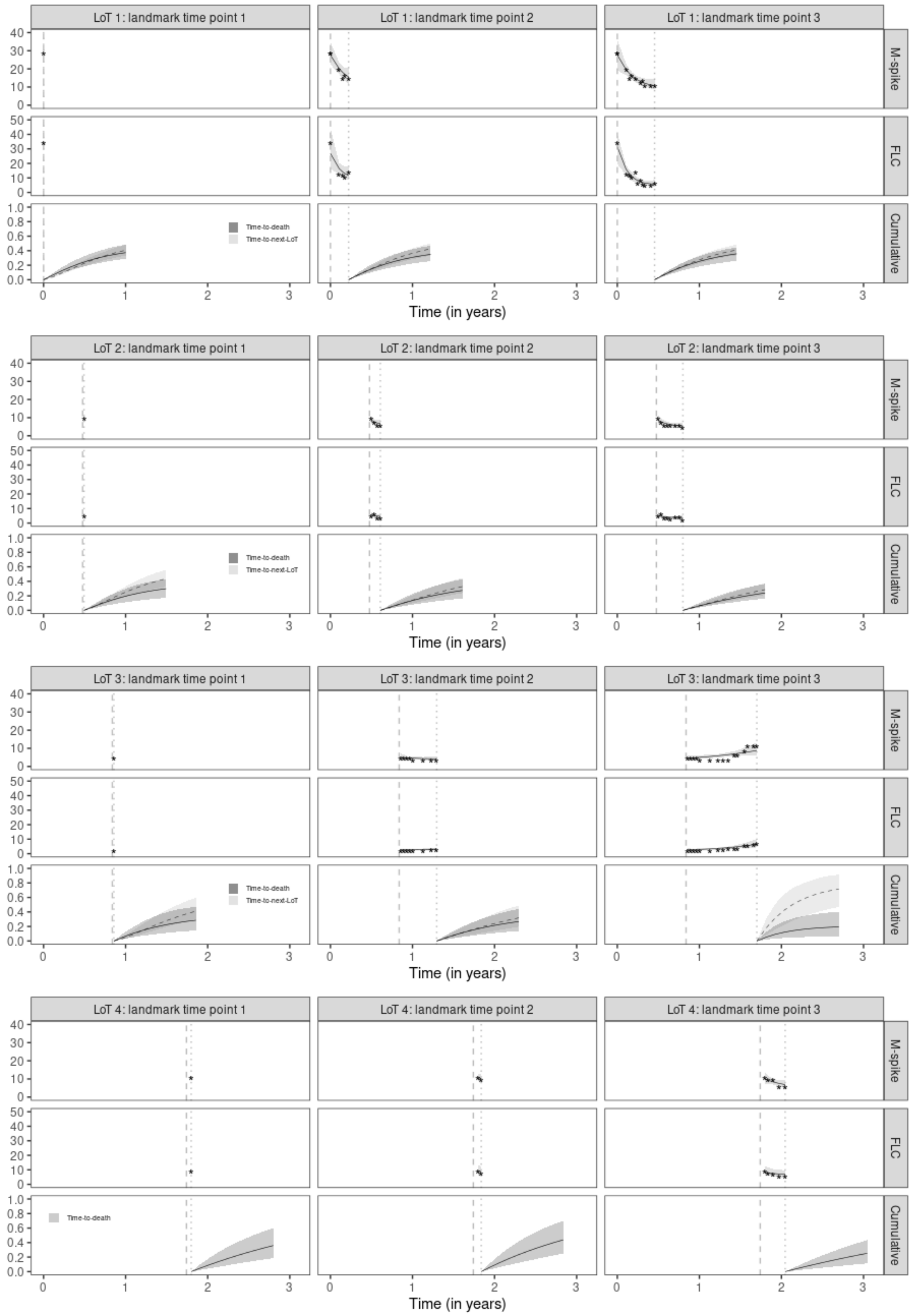

Web Figure 7: One-year dynamic predictions from three landmark time points (vertical dotted lines) in each line of therapy (LoT) using the joint estimation (JE) approach for patient A. Vertical dashed lines indicate LoT initiation times. For "M-spike" and "FLC" rows, stars represent biomarker observed values with their respective posterior mean trajectory (solid lines) and 95% credible intervals (gray shadow). For "Cumulative" rows, solid and dashed lines are posterior means (with their respective 95% credible intervals) of cumulative incidence functions (LoTs 1, 2 & 3) or distribution functions (LoT 4) for time-to-death and time-to-next-LoT, respectively.

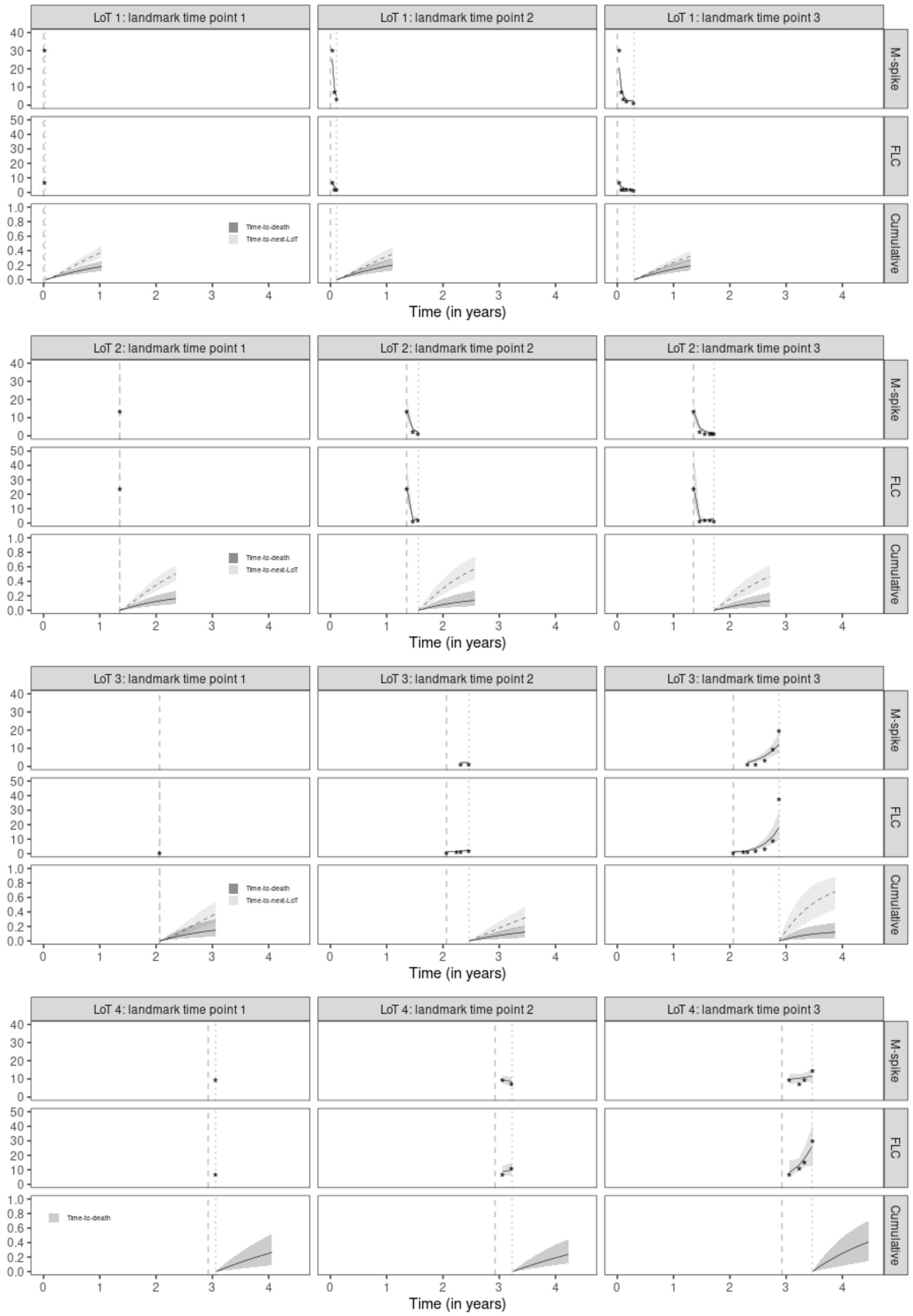

Web Figure 8: One-year dynamic predictions from three landmark time points (vertical dotted lines) in each line of therapy (LoT) using the joint estimation (JE) approach for patient B. Vertical dashed lines indicate LoT initiation times. For "M-spike" and "FLC" rows, stars represent biomarker observed values with their respective posterior mean trajectory (solid lines) and 95% credible intervals (gray shadow). For "Cumulative" rows, solid and dashed lines are posterior means (with their respective 95% credible intervals) of cumulative incidence functions (LoTs 1, 2 & 3) or distribution functions (LoT 4) for time-to-death and time-to-next-LoT, respectively.
